# Comparative transcriptomics and proteomics of three different aphid species identifies core and diverse effector sets

**DOI:** 10.1186/s12864-016-2496-6

**Published:** 2016-03-02

**Authors:** Peter Thorpe, Peter J. A. Cock, Jorunn Bos

**Affiliations:** Cell and Molecular Sciences, The James Hutton Institute, Invergowrie, Dundee, DD2 5DA UK; Information and Computational Sciences, The James Hutton Institute, Invergowrie, Dundee, DD2 5DA UK; Dundee Effector Consortium, The James Hutton Institute, Invergowrie, Dundee, DD2 5DA UK; College of Life Sciences, University of Dundee, Dundee, UK

**Keywords:** Aphid, Effector, Host-range, RNA-seq, Proteomics

## Abstract

**Background:**

Aphids are phloem-feeding insects that cause significant economic losses to agriculture worldwide. While feeding and probing these insects deliver molecules, called effectors, inside their host to enable infestation. The identification and characterization of these effectors from different species that vary in their host range is an important step in understanding the infestation success of aphids and aphid host range variation. This study employs a multi-disciplinary approach based on transcriptome sequencing and proteomics to identify and compare effector candidates from the broad host range aphid *Myzus persicae* (green peach aphid) (genotypes O, J and F), and narrow host range aphids *Myzus cerasi* (black cherry aphid) and *Rhopalosiphum padi* (bird-cherry oat aphid).

**Results:**

Using a combination of aphid transcriptome sequencing on libraries derived from head versus body tissues as well as saliva proteomics we were able to predict candidate effectors repertoires from the different aphid species and genotypes. Among the identified conserved or core effector sets, we identified a significant number of previously identified aphid candidate effectors indicating these proteins may be involved in general infestation strategies. Moreover, we identified aphid candidate effector sequences that were specific to one species, which are interesting candidates for further validation and characterization with regards to species-specific functions during infestation. We assessed our candidate effector repertoires for evidence of positive selection, and identified 49 candidates with DN/DS ratios >1. We noted higher rates of DN/DS ratios in predicted aphid effectors than non-effectors. Whether this reflects positive selection due to co-evolution with host plants, or increased neofunctionalization upon gene duplication remains to be investigated.

**Conclusion:**

Our work provides a comprehensive overview of the candidate effector repertoires from three different aphid species with varying host ranges. Comparative analyses revealed candidate effectors that are most likely are involved in general aspects of infestation, whereas others, that are highly divergent, may be involved in specific processes important for certain aphid species. Insights into the overlap and differences in aphid effector repertoires are important in understanding how different species successfully infest different ranges of plant species.

**Electronic supplementary material:**

The online version of this article (doi:10.1186/s12864-016-2496-6) contains supplementary material, which is available to authorized users.

## Background

Aphids are phloem-feeding insects that cause substantial damage to agriculture worldwide due to feeding-related damage and the transmission of economically important plant viruses [1]. Effective control of aphids in field crops currently relies heavily on the use of insecticides. However, aphids have been shown to develop resistance to many of the different types of available insecticides [2–4]. In addition, there are an increasing number of restrictions in place on the use of insecticides under EU legislation due to their environmental impact [5]. Therefore, there is a pressing need to develop novel aphid control strategies, which requires a better understanding of the molecular basis of plant-aphid interactions.

Among the over 4000 aphid species, around 10 % are considered pests of economically important plants and trees [6]. While most aphid species are highly specialized and can only infest plants in a single taxonomic family or several related plant species, some aphid species have an exceptionally broad host range and are able to infest plants in many families [1]. The latter group of aphid species includes some major pests, like *Myzus persicae* (green peach aphid), which infests plants in over 40 families, including crops like potato and oil seed rape [1]. In contrast, a close relative of *M. persicae*, *M. cerasi* (black cherry aphid), is only able to infest cherry and a few herbaceous plants. Also, some aphids, like *Rhopalosiphum padi* (bird cherry-oat aphid), mainly infest cereals. Interestingly, we previously showed that aphid species *M. persicae*, *M. cerasi* and *R. padi* exhibited probing behaviour on *Arabidopsis thaliana* during host, poor-host as well as nonhost interactions [7]. This implies that during these different types of interactions there is an opportunity for molecular interactions to take place. Moreover, we found that *Arabidopsis* transcriptional responses to these three aphid species showed a high level of overlap, suggesting that also aphid responses likely play a key role during the different types of interactions. Although the molecular mechanisms underlying aphid host range differences remain elusive, it is likely both plant and aphid molecules are involved [8].

For a plant pathogen or pest to be successful on a host, it is important to manipulate host cell processes to promote virulence. This generally involves the secretion of molecules, termed effectors, inside the host, which target host molecules [9]. A number of recent studies have now shown that insects, including aphids, produce and secrete effectors that suppress or induce plant defence responses [10–13]. These aphid effectors are thought to be produced predominantly in the salivary glands and secreted within aphid saliva during probing and feeding [14–19]. The recent availability of aphid genome and transcriptome sequence data has facilitated the development of approaches to identify aphid candidate effectors [10, 11, 20–22]. More specifically, bioinformatic pipelines to identify putative secreted proteins have been developed e.g. [23] and applied to several aphid species [10, 11–20]. In addition, saliva collection methods based on artificial diet-feeding systems in combination with mass spectrometry have allowed the identification of proteins present in saliva of several aphid species [10, 21, 24]. These efforts have generated lists of candidate effector proteins for a number of species and led to the functional characterization of several candidates in plant-aphid interactions.

We were interested to gain a comprehensive insight into the diversity of aphid effector repertoires of species with varying host ranges. Therefore, we employed a combined transcriptomic and saliva proteomic approach to identify and compare the effector repertoires from three different aphid species, *M. persicae*, *M. cerasi* and *R. padi*. For *M. persicae*, we included three different genotypes to also assess variation within this species. These were genotype O, which is currently most prevalent in the UK, genotype J which was prevalent in the UK around 1970 but is currently only found occasionally, and genotype F, which was prevalent in 1995 but is not currently found (Brian Fenton, personal communication, 2015). These genotypes show differences in growth rates on different host species, with genotype F showing a significantly slower growth on all host species compared to other genotypes [25].

We found a large number of predicted secreted aphid proteins to be highly conserved among the different aphid species, which we propose reflects the potential aphid core effector repertoire. Many proteins within this repertoire were predicted to be of unknown function and specific to aphids. Therefore, these proteins may exhibit highly conserved functions important in establishing plant-aphid interactions. In addition, we identified sets of effectors that were highly divergent among the different aphid species and/or genotypes, as well effectors potentially specific to one of the aphid species. Some of these effectors showed evidence of positive selection. We propose that such effectors are strong candidates for contributing to aphid species-specific infestation strategies.

## Results and discussion

### De novo RNA-seq data assembly

**Fig. 1:**
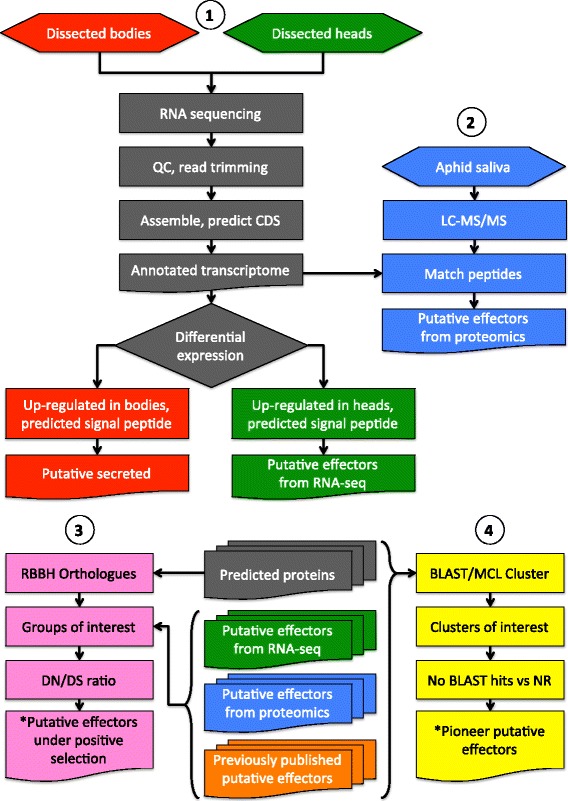
Diagrammatic representation of the experimental procedure used to identify putative effectors from *Myzus persicae* genotype O, J and F, *M. cerasi* and *Rhopalosiphum padi*. (*1*) Aphids were dissected into biological replicas of heads and separately bodies (without nymphs). RNA was extracted and subjected to Illumina HiSeq sequencing. Following quality control (QC) and assembly, differential expression was performed to identify transcripts upregulated in head samples that encoded predicted signal peptides. These were categorised as putative effectors. (*2*) Aphid saliva was collected in artificial feeding chambers. The saliva was subjected to LC-MS/MS analysis. The resulting data was interrogated against the transcriptome assemblies in order to identify salivary secreted proteins. These were categorised as putative effectors. (*3*) Reciprocal best BLAST hit analysis was used to identity 1:1 ratio orthologues between *M. persicae* genotype O, J and F, *M. cerasi*, *R. padi*, *Acyrthosiphon pisum* and *Aphis glycines.* Clustering of the 1:1 ratio orthologous sequences was performed and where the resulting orthologous clusters contained a putative effector, they were subjected to DN/DS analysis. Clusters with a DN/DS value greater than 1 were identified as potentially under selection pressure. (*4*) Whole transcriptome clustering based on sequence similarity using BLAST and MCL, using the species listed above including *Drosophila melanogaster*, was used to identify clusters of putative effectors and those which maybe novel, termed pioneers in this study

To define the effector repertoires from aphid species *M. persicae* (genotypes O, J and F), *M. cerasi* and *R. padi*, we sequenced libraries generated using RNA extracted from both body and head tissues. Quality control and *de novo* assembly was performed for each species and genotype. We performed differential gene expression analyses by mapping reads for each biological replicate dataset back to the assemblies and then generated normalised digital gene expression (TMM-FPKM) (Fig. 1). Details on the numbers of assembled contigs, reads, predicted coding sequences (CDS) and differentially expressed genes are summarised in Table 1.

**Table 1:** Statistics, number of differentially expressed transcript and predicted effectors for the *de novo* RNA-seq assemblies generated in this project for *Myzus persicae* genotype O, J and F, *M. cerasi* and *Rhopalosiphum padi*

	*M. cerasi*	*R. padi*	*M. persicae* genotype O	*M. persicae* genotype F	*M. persicae* genotype J
Unigenes from CDS	28,408	28,542	23,822	24,742	21,441
Transcripts:	126,245	35,426	125,222	122,733	108,577
Components (genes):	60,095	32,357	62,850	63,350	55,644
Percent GC:	34.6	36.3	34.6	34.5	34.3
Total assembled bases:	193,365,154	22,967,672	192,529,031	168,958,624	164,626,862
Upregulated head	1410	950	1370	3762	1383
Secreted	144	165	276	541	355
Secreted w/NLS	12	18	45	84	64
Upregulated bodies	848	893	796	2575	2692
Secreted	64	94	133	252	278
Secreted w/NLS	3	10	9	25	28

Predicted unigenes from the *de novo* assemblies were subjected to BLAST searches against the NCBI NR database (March 2014) to annotate the transcript coding sequences (CDS) and identify potential contaminants through kingdom assignment. In summary, the *de novo R. padi* RNA-seq assembly resulted in the prediction of 28,542 CDS, of which 1189 did not have any BLAST hit (1e-5 threshold) (Table 1). The majority (91 %) of CDS showed similarity to *Acyrthosiphon pisum* (pea aphid), for which the genome sequence is available [26]. Only 2 sequences were identified as viral, and matched to putative replicase proteins from the insect virus *Euprosterna elaeasa*, and 29 sequences were bacterial, including 2 sequences from the aphid endosymbiont *Buchnera*. The number of transcripts assembled for *R. padi* was substantially less than that of the other assemblies, likely due to the lower number of reads generated for this species. For *M. cerasi* we identified 28,408 unigene CDS, with 76 % showing BLAST hits to *A. pisum*. The *M. cerasi* CDS set included 18 viral sequences, and 183 bacterial sequences of which 120 were *Buchnera*-derived. For the *M. persicae* genotypes the numbers of CDS ranged from 21,441 to 24,742, with around 90 % showing BLAST hits against *A. pisum*. The number of sequences with similarity to insect viruses ranged from 8 to 11, and the number of bacterial sequences ranged from 114 to 228, with 73 to 164 being derived from *Buchnera*. We also identified a number of transcripts that showed BLAST hits to plant genes (114 for *R. padi*, 56 for *M. cerasi*, 43 for *M. persicae* genotype J, 24 for *M. persicae* genotype F, and 27 for *M. persicae* genotype O). However, it is unclear whether these transcripts are incorrectly annotated in the database or whether they are indeed present in insect tissue. We also found a number of secondary BLAST hits against *Clostridium sordellii* sequences from one particular dataset (ATCC 9714) [27], which included hits for some well characterized aphid effectors, such as MpC002 and Me10. However, additional BLAST searches showed that these aphid effectors do not show any hits against other bacterial databases, including other *Clostridium* datasets. Also effectors like C002 contain introns and are confirmed to be aphid derived using various independent approaches, including proteomics and genomics [17, 20–22]. The relatively high number of matches to a specific *C. sordellii* database is therefore unlikely due to contamination of our samples.

**Fig. 2:**
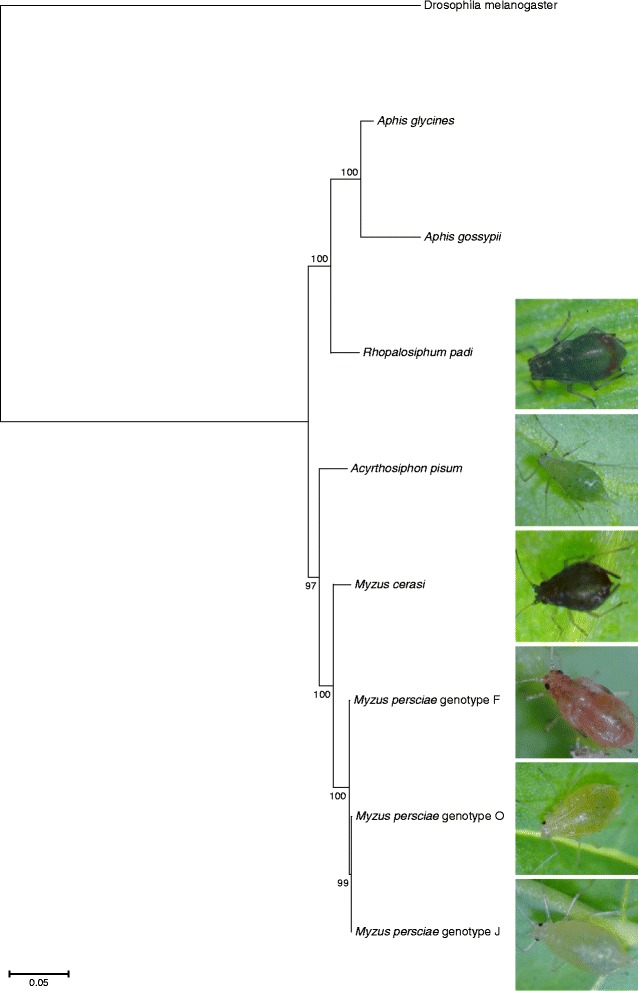
Phylogenetic tree from maximum likelihood analysis of an alignment of 71 EOG genes per species using 150 bootstraps

We used our *de novo* transcriptome datasets as well as the *A. pisum* genome sequence, and the publicly available transcriptome datasets for *Aphis glycines* (soybean aphid) [28] and *A. gossypii* (melon aphid) [29], for phylogenetic analyses to assess the relationships of the different aphid species used in this study. We selected a set of single copy orthologous genes described by Misof et al. [29] for reciprocal best BLAST hit and phylogenetic analyses (Additional file 1). As expected the two different *Myzus* species were more closely related to each other than to the other species in the phylogenetic tree (Fig. 2). Also, the three *M. persicae* genotypes clustered together, with genotype O being phylogenetically closer to genotype J than genotype F (Fig. 2).

In addition, we looked at the predicted functions of the most abundant transcripts for each aphid species and genotype. Annotation of the 100 most abundant transcripts in the (combined head and body) transcriptome datasets revealed a high level of similarity across the species/genotypes (Additional file 2). For example ribosomal proteins, cuticle proteins, ATP synthases, elongation factor 1-alpha, myosin light chain protein, putative sheath protein, chemosensory proteins, and heat shock-associated proteins were present in the 100 most abundant transcripts for all species. Interestingly, we identified transcripts with sequence similarity to several previously identified putative aphid effectors among the 100 most abundant transcripts. For most aphid species/genotypes we identified transcript sequences with similarity to effector Me10 from *Macrosiphum euphorbiae* (potato aphid), which has previously been shown to promote aphid virulence [10]. Moreover, we also identified several transcripts in the various datasets with similarity to candidate effectors, including Mp10 (*M. cerasi*, *M. persicae* genotype O and F), Mp12 (*M. persicae* genotype F and O), Mp17 (*M. persicae* genotype O), Mp23 (*M. persicae* genotype O, F and J), Mp44 (*M. persicae* genotype F), and MpC002 (*M. cerasi*) [22]. The high level of overlap in predicted functions of the most abundant transcripts for the different aphid species reflects their importance to aphid biology. Although we identified transcripts with similarity to several known putative effectors for the *Myzus* species, we only identified one previously predicted effector sequence (similar to Me10) in the *R. padi* top 100 transcript set. It is possible this aphid species utilizes a different effectors repertoire than *Myzus* species during plant infestation.

### Prediction of putative effector repertoires from different aphid species

Effectors are thought to be expressed and synthesised in the salivary glands, which are located in the aphid head [17]. Aphid species vary in size and therefore dissection of salivary gland tissues to use for transcriptome analyses is challenging and not always feasible. To be able to identify transcripts encoding putative effectors for the different aphid species, which are most likely expressed in the salivary glands, we compared datasets generated from aphid head versus body tissues and looked for transcripts that were up-regulated in head tissue.

First, we identified transcripts up-regulated in head versus body tissues and vice versa for each species (Table 1). Then, we applied a bioinformatics pipeline to these transcript sets to identify transcripts up-regulated in aphid heads that are predicted to encode secreted proteins. This identified 144 transcripts for *M. cerasi*, 165 for *R. padi*, 276 for *M. persicae* genotype O, 541 for *M. persicae* genotype F, and 355 for *M. persicae* genotype J (Table 1; Additional file 3). In addition we predicted the presence of nuclear localization signals (NLS), which identified 12 to 84 predicted secreted proteins with a predicted nuclear localization (Table 1). Interestingly, these results show variation in numbers of predicted effectors within *M. persicae*. For example, for genotype F we nearly predicted twice the number of effectors as compared to genotype O despite similar numbers of total CDS. Whether these differences are due to the quality of the assembly or reflect any biological relevance remains to be investigated.

In parallel, we examined the gene expression profiles of a set of genes commonly used for normalisation of qPCR data and thought to be constitutively expressed [30, 31]. Sequences annotated as elongation factor 1 alpha, actin, succinate dehydrogenase and CDC42 (cell division control protein) were selected for assessment of their expression profiles. Of the 128 aphid gene sequences we identified using sequence similarity searches to these select genes, only 4 were found to be differentially expressed (Additional file 4). One was an actin transcript, and the other three were succinate dehydrogenase transcripts. However, two out of the three differentially expressed succinate dehydrogenase genes were only expressed, to low levels, in one of the aphid samples (*M. persicae* genotype F - head) with no expression in the other samples, thus leading to differential expression (Additional file 4). Some actin genes could be affected in their expression by differences in aphid growth. Similarly, actin was highly expressed in J2 nematodes versus those in other life stages in the case of *Globodera pallida* [32]. Despite this, 124 of the 128 transcripts did not show any differential gene expression within our dataset indicating that these genes are not regulated similarly to those in our candidate effector sets.

Previous studies have identified salivary proteins in aphids using proteomics and/or bioinformatics approaches [10, 11, 20, 21, 33]. We compared the predicted aphid effectors identified by Bos et al. [11], Atamian et al. [10], and Carolan et al. [20] to the datasets we generated in this study and found similar sequences for many of the previously identified candidate effectors (Additional file 3). For example, we identified sequences similar to previously identified effectors C002 [17] and Me10 [10], to the candidate effectors identified by Bos et al. [22] and to 276 candidate effectors described by Carolan et al. [20] based on a BLASTP cut-off of 1e-10 (Additional file 3).

GO annotations were assigned and GO enrichment analyses revealed an over-representation in the predicted effector repertoires of all species of the functional categories “structural molecule activity” and “constituent of cuticle and extracellular region” (Additional files 5 and 6). However, many putative effectors identified to date from different pathosystems have no known GO domains [34]. Therefore these GO-term data may not reflect the biological function of a large number of aphid predicted effectors.

We then looked at BLAST similarity searches of our predicted effector repertoires to look at putative functions (Additional file 3). Interestingly, we identified two predicted aphid effectors from *M. persicae* with similarity to a pea aphid E3 ubiquitin ligase [GenBank: XP_001945627.1]. These enzymes are important components of the ubiquitin-proteasome pathway. The ubiquitin-proteasome pathway is implicated in a wide range of plant-pathogen interactions and it is possible that aphids exploit this pathway in order to manipulate host responses [35, 36].

Also, we identified putative effectors with potential roles in detoxification and digestion (Additional file 3). It has been suggested that aphids secrete cellulase enzymes in order to minimise the mechanical damage caused during stylet movement [15]. We did not find cellulase enzymes or any other glycosyl hydrolase (GH) domain containing protein involved in cell wall degradation in our predicted effector repertoires. However, when interrogating the whole transcriptome, rather than the effector repertoire sets, we found 10 transcripts corresponding to GH5 domain cellulases (*M. persicae* and *M. cerasi* only), of which 8 were predicted to encode secreted proteins. However, these transcripts were similarly expressed in body and head tissues. It is possible that some effectors may be produced in other tissues than the salivary glands and are then transported to the salivary duct and secreted into saliva. Therefore, our selection of candidate effectors based on high expression levels in head versus body tissues may have missed some potential effectors of interest. Moreover, secreted enzymes in the aphid digestive tract are likely involved in the detoxification and degradation of plant compounds [37, 38].

Carolan et al. [20] previously identified some similarity between nematode effectors and predicted pea aphid effectors based on functional annotations. We assessed our datasets for such similarity and identified sequences with predicted functions similar to those of several root knot nematode effectors (i.e. m1 zinc metalloprotease, calreticulin and glutathione peroxidase) (Additional file 3). In addition, we compared amino acid sequences within our candidate effector sets to the predicted effector repertoire of the cyst nematode *Globodera pallida* [34]. We only found *M. persicae* sequences with similarity to three proteins, which are potentially part of a family [GeneDB/WormBase: GPLIN_001205000, GPLIN_000990400, GPLIN_000574800] and are predicted to encode a gland cell secretory protein 3, which contains a thioredoxin-like domain, and shows similarity to a protein with a kinase domain [GeneDB/WormBase: GPLIN_000510600] (BLASTP e-value *p* < 1e-21). Overall, there is very little, if no, convergent evolution between the effectors repertoires of the aphid species used in this study and those of plant pathogenic nematodes.

We also compared our findings to transcriptome studies aimed at identifying salivary gland genes from other sap-sucking insects within the order Hempitera, such as the potato leafhopper *Empoasca fabae* [39], the whitefly *Bemisia tabaci* [40], and the brown planthopper *Nilaparvata lugens* [41]. Genes with functions predicted to be involved in plant-hemipteran interactions, that may have similar roles to those identified in this study, include peroxidases [42], sucrase [43], peptidase, lipase [39, 44], phosphatase [45], glucose dehydrogenase [45] and a number of hypothetical pea aphid proteins. Previously it has been shown that some predicted secretory salivary proteins from the whitefly show similarity to putative pea aphid effectors [40]. For example, predicted whitefly effectors showed similarity to an GMC oxireductase, glucose dehydrogenase, Mp12, Mp43, Mp11, Mp43, Mp46, sucrase, and M1 zinc metalloprotease [40]. Moreover, comparative analyses between a planthopper salivary gland transcriptome and the pea aphid identified number of similar sequences which may function in insect-plant interactions such as a glucose dehydrogenase, peroxidase-like, vitellogenin-6-like, serine protease snake-like isoform 1 carboxypeptidase, and digestive enzymes [41]. This shows that these insects may use some common proteins in order to successfully infest their hosts. However, a large number of aphid putative effectors identified here are aphid specific, consistent with previous research [20], indicating aphid specific evolution.

### Prediction of putative effectors using saliva proteomics

Complementary to our transcriptomics approach we performed aphid saliva proteomics for the three aphid species/genotypes in our study to identify candidate effectors. We collected saliva using an artificial feeding system [21, 22] and subjected samples to LC-MS/MS analyses. MASCOT software searches for peptide identification were run against the *de novo* assemblies generated in this project (Fig. 1). In total we identified 56 proteins in the saliva of *R. padi*, 19 proteins in the saliva of *M. cerasi*, and 40, 42 and 47 proteins in saliva of *M. persicae* genotypes F, O and J, respectively (Additional file 7). The differences in protein numbers could reflect that these aphid species produce variable amounts of saliva when exposed to artificial feeding system or that they secrete effector repertoires with different complexities.

Also, we also performed MASCOT searches using the NCBI NR database, which led to a relatively small number of proteins being identified when compared using the *de novo* assemblies (Additional file 7). More specifically, we only identified 10 proteins for *R. padi*, 2 for *M. cerasi*, 6 for *M. persicae* genotype F, 15 for *M. persicae* genotype O, and 12 for *M. persicae* genotype J. This highlights the importance of generating *de novo* transcriptomes for different species in applying a proteomics approach for protein identification.

Out of 204 proteins identified for the different species and genotypes in total, only 61 contained predicted signal peptides with no transmembrane domain. Nineteen of these were in our predicted aphid effector datasets based on RNA-seq analyses. Overall this shows that based on our analyses less than one third of the 204 proteins contain secretion signals, and that only a small number of candidate effectors were identified by both the proteomics and transcriptomics approach. When assessing the gene expression profiles of the candidate effectors identified by proteomics we found that the majority of corresponding transcript where more highly expressed in head versus body tissue (Additional file 8). However, when applying statistical analyses we found that for only 63 out of the 204 proteins the transcripts were significantly more abundant in head tissues. Lack of differential expression therefore partly explains the lack of correlation between our effector sets defined by RNA-seq and proteomics approaches.

Another reason for the lack of overlap could be missing 5′-sequences in our transcriptome dataset required for prediction of signal peptide sequences. We performed an overall assessment of the *de novo* assemblies for full-length transcripts using TransDecoder. Out of 28,542 *R. padi* predicted transcripts only 4590 were predicted to be complete (16 %), 14,505 were internal (50 %), 6306 were 5′-partial (22 %), and 2241 were 3′-partial (7.6 %). Similar numbers were obtained for the other aphid species and genotypes. In addition, we assessed whether full-length transcript sequences were available for the 204 proteins identified by proteomics within the *de novo* assemblies generated for each species. For the 56 *R. padi* proteins identified by proteomics, we only found full-length transcript data for 8 proteins, which limited our ability to predict signal peptide sequences. Twelve out of the 56 proteins were predicted to contain a signal peptide, with 4 of these containing transmembrane domains. For *M. cerasi*, full-length transcripts were available for 12 out of 19 proteins identified by proteomics. Ten proteins contained signal peptide sequences, of which 2 contained transmembrane domains. For the *M. persicae* genotypes we found that full-length transcripts were available for about 50 % of the proteins identified by proteomics.

We then compared functional annotations of saliva proteins from the different aphid species. We found that a putative sheath protein [GenBank: AFT82624.1], several uncharacterized proteins [GenBank: XM_003246795.2, XM_003246613.2, XM_008184371.1 and XM_003242933.2], and a peroxidase-like [GenBank: XP_003247027.1], were secreted by all aphid species. Whereas uncharacterized protein [GenBank: XM_008184371.1] and trehalase-like isoform X1 [GenBank: XP_003245895.1] were found in saliva from all *Myzus* species, including all genotypes. Glutathione S-transferase [GenBank: XP_001942714.1] and uncharacterized protein [GenBank: NM_001162275.2] were identified only in saliva from the different *M. persicae* genotypes. Twenty-two proteins were found only in saliva from *R. padi* such as C002, nine hypothetical/unknown proteins, carbonic anhydrase and proteins with proteolytic activity such as aminopeptidase and cathepsin B-348 (Additional file 7). It is possible that this aphid secretes different effectors into the artificial diet, but we cannot rule out that observed differences are due to differences in saliva amounts secreted and quality of the RNA-seq datasets used for identification of peptides.

Although, we cannot draw conclusions regarding the presence or absence of certain proteins in saliva of specific species due to the lack of biological and technical replication in our experimental set-up, our data does support a model wherein different aphids secrete a core or common effector set inside their host to manipulate host processes. Within the common set of secreted saliva proteins, those predicted to encode enzymes may be involved in detoxification of chemical defences compounds induced during early plant defence responses to reduce harmful levels of reactive oxygen species [9]. Detoxification of plant defence responses may be a common strategy employed by aphids and identifying and characterizing any common/core proteins involved in this could provide novel broad range targets for aphid control strategies.

### Cluster analysis to identify core effector sets

One of our key interests in this study was to compare the predicted aphid effector repertoires to identify common or core sets of candidate effectors as well as those potentially unique to specific species and/or genotypes, or highly divergent across species. To do this, we used the transcriptome and proteomics datasets for *R. padi*, *M. cerasi*, and *M. persicae* generated here in combination with publicly available sequence data sets for *A. pisum* [26], *A. glycines* [28], as well as the fruit fly *Drosophila melanogaster* [46], for cluster analyses. The transcriptome of *A. glycines* was re-assembled for our study.

To perform cluster analysis based on sequence similarity, a database of all amino acid sequences from all species listed above was generated, including several previously published candidate effector sets [11, 12, 20]. This amino acid database was subjected to a self-BLASTP (evalue 1e-35) similarity search followed by cluster analyses using MCL (Fig. 1). Clusters containing any of the candidate effectors identified by our transcriptomics or proteomics approach or previously reported [11, 12, 20] were defined as candidate effector containing-clusters. We identified 444 candidate effector containing-clusters represented by 6652 sequences out of the total 43,256 clusters represented by 216,403 sequences. Within the candidate effector containing-clusters we looked for those that were represented by 5 of the 8 aphid datasets, and defined these as core effectors (Additional file 9). This identified 199 core putative effector clusters containing 4811 sequences (Additional file 10). Similarity searches revealed that these core putative effectors showed high similarity to proteins with a range of different functions, such as a glucose dehydrogenase, sheath protein, apolipophorin precursor as well as previously reported aphid candidate effectors of unknown function (Additional file 9).

Many of the predicted core effectors encode enzymes with predicted functions in detoxification or digestion. However, we also identified predicted effectors that have no sequence similarity to proteins of known functions, of which some are aphid-specific. Importantly, our cluster analyses revealed similarity of core effectors to a significant number of previously identified candidate effectors. These include *M. persicae* effector Mp10, which triggers a range of plant defences and reduces aphid virulence when over-expressed *in planta* [47]. Two clusters contained effectors with known virulence activity, MpC002 and Me10. C002 is one of the best-characterized aphid effectors and the *M. persicae* form of C002, called MpC002, contributes to aphid virulence as shown by a combination of *in planta* overexpression and RNAi experiments [11, 13]. In addition, the *M. euphorbiae* effector Me10 enhances aphids virulence upon *in planta* over-expression [10].

### Identification of potential aphid species-specific candidate effectors

In addition to the conserved effectors, we also looked for any MCL clusters that contained candidate effector sequences with no BLAST hit against the NCBI NR database (e-value 1e-5) or Pfam A domains specific to a single aphid species or genus. These were defined as pioneer candidate effectors. We found 7 clusters corresponding to 10 sequences specific to *R. padi*, and 8 clusters corresponding to 11 sequences specific to *M. persicae*. We found 4 clusters, containing 16 sequences that were specifically represented by the two *Myzus* species (Additional file 10). These pioneer candidate effectors were all predicted based on our transcriptomics analyses and therefore showed significantly higher expression in head samples than in body samples (*p* < 0.001).

To determine whether these potentially species-specific effectors are indeed species-specific further characterization will be required. This will address whether *M. persicae*-specific effectors contribute to host range and whether the cereal pest *R. padi* requires specific effectors to successfully infest cereals.

### DN/DS analysis identifies candidate effectors under positive selection

In addition to species-specific effectors, aphids may secrete different variants of effectors involved in host interactions. For example, some aphid effectors may have evolved and exhibit diversity reflecting co-evolution of specific plant and aphid species. To determine if the putative effectors identified in this study were under selection pressure, the ratio of the number of nonsynoymous substitutions to the number of synonymous substitutions per synonymous site (DN/DS) was calculated for reciprocal best blast (RBBH) hit orthologous groups (Fig. 1). First, we generated a reciprocal best BLAST hit putative 1:1 orthologous group network, by performing reciprocal best blast hit analyses (BLASTP) using the different aphid transcriptome datasets (including the pea aphid predicted genes). A network was generated from the resulting hits (Additional file 10). We calculated DN/DS ratios for each RBBH group containing a putative effector and identified those that scored a DN/DS ratio >1, indicative of positive selection (Fig. 1; Table 2). Since our analysis is based on transcriptome rather than genome sequencing data, we were unable to take potential gene duplication into consideration, which is known to occur within aphid genomes [26]. However, previously identified effectors such as C002, Mp1 and Me10 are single copy genes based on BLAST searches against the published pea aphid genome.

**Table 2:** Reciprocal best blast hit analysis identified 1:1 orthologues between the transcriptomes. The resulting clusters, if they contained a putative effector were subjected to DN/DS analysis to identify any clusters under positive selection (DN/DS >1.0). Those identified as possibly under selection are listed in the table

Putative annotation	Identified by	DN/DS	Cluster number	Species in cluster
Uncharacterized protein LOC100570454	Proteomics	4.17	12679	*M. persicae*
Twitchin-like *A. pisum*	Proteomics	3.66	11555	*M. persicae*
Uncharacterized protein LOC100160301	Proteomics, Mp15 (Bos et al., [14])	3.62	11125	*M. persicae*
A-agglutinin anchorage subunit-like *A. pisum*	Proteomics	2.93	4788	*Myzus*
Carbonic anhydrase 7-like *A. pisum*	Proteomics	2.79	4096	*Myzus*
Hypothetical protein LOC100574284	Proteomics	2.48	12749	*M. persicae*
Peroxidase-like, partial *A. pisum*	Proteomics	2.32	9497	*M. persicae*
Uncharacterized protein LOC100167427 precursor *A. pisum*	Proteomics, Me10 (Atamian et al., [11])	1.90	3800	*Myzus*, *A. pisum*, *R. padi*
Carbonic anhydrase 7-like *A. pisum*	Proteomics	1.43	10713	*M. cerasi*, *A. pisum*, *R. padi*
Glucose dehydrogenase acceptor-like *A. pisum*	Proteomics	1.24	12369	*M. persicae*
Hypothetical protein LOC100159010 *A. pisum*	Proteomics	1.24	14505	*M. persicae*, *R. padi*, *A. glycines*
Carbonic anhydrase 7-like A. pisum	Proteomics, Mp50 (Bos et al., [14]	1.15	6	*Myzus*, *A. pisum*, *R.padi*
Uncharacterized protein LOC100575478 precursor *A. pisum*	Mp35 (Bos et al., [14])	2.99	3804	*Myzus*
ACYPI43360 *A. pisum*	Mp31 Bos et al., [14]	2.64	1099	*Myzus*, *A. pism*
Hypothetical protein LOC100167863 *A. pisum*	MpCOO2 (Bos et al., [14])	2.63	3810	*Myzus*
Hypothetical protein LOC100569335 *A. pisum*	Mp6 (Bos et al., [14])	1.90	1095	*Myzus*
Hypothetical protein LOC100159632 *A. pisum*	Carolan et al., (2011)	1.47	6601	*M. persicae*, *R. padi*
Protein takeout-like *A. pisum*	Mp12 (Bos et al., [14])	1.48	3497	*M. persicae*, *A. pisum*
Uncharacterized protein LOC100159485 precursor *A. pisum*	Mp54 (Bos et al., [14])	1.15	1096	*Myzus*
Mitochondrial import inner membrane translocase subunit	Carolan et al., [23]	2.32	6744	*M. persicae*
Superoxide dismutase Cu-Zn-like precursor *A. pisum*	Carolan et al., [23]	2.02	569	*M. persicae*, *R. padi*, *A. glycines*
Sarcalumenin-like isoform X1 *A. pisum*	Carolan et al., [23]	1.34	244	*Myzus*, *A. pisum*, *R. padi*, *A. glycines*
LOC100167075	Carolan et al., [23]	1.14	536	*Myzus*, *A. pisum*, *A. glycines*
Cuticular protein 62 precursor *A. pisum*	Carolan et al., [23]	1.13	6596	*M. persicae*, *A. pisum*, *A. glycines*
LOC100163954	Carolan et al., [23]	1.05	6586	*M. persicae*, *A. pisum*
Pioneer	Bioinformatics	1.83	12331	*M. pericae*
LOC100162609 *A. pisum*	Bioinformatics	1.83	12665	*M. pericae*
LOC100571623 *A. pisum*	Bioinformatics	1.78	1548	*Myzus*, *A. pisum*
LIRP-like *A. pisum*	Bioinformatics	1.69	1457	*Myzus*, *A. pisum*
ACYPI000490 *A. pisum*	Bioinformatics	1.68	13765	*M. persicae*, *A. pisum*
Peroxidase-like	Bioinformatics	1.67	6520	*Myzus*, *A. pisum*
LOC100570826 *A. pisum*	Bioinformatics	1.65	9610	*Myzus*
Odorant-binding protein	Bioinformatics	1.63	3530	*Myzus*, *A. pisum*, *R. padi*
Cuticle protein	Bioinformatics	1.63	4482	*Myzus*, *A. pisum*
Zinc finger protein	Bioinformatics	1.61	12433	*M. persicae*
gi|488530945	Bioinformatics	1.56	5718	*Myzus*
LOC100570068 *A. pisum*	Bioinformatics	1.43	4578	*Myzus*
Pioneer	Bioinformatics	1.42	6006	*Myzus*
LOC100163563 *A. pisum*	Bioinformatics	1.35	923	*Myzus*, *A. pisum*
LOC100162393 *A. pisum*	Bioinformatics	1.35	245	*Myzus*, *A. pisum*, *R. padi*
LOC100169018 *A. pisum*	Bioinformatics	1.30	4753	*M. persicae*
Pioneers	Bioinformatics	1.29	4395	*Myzus*
LOC100160479 *A. pisum*	Bioinformatics	1.24	3806	*Myzus*, *A. pisum*
LOC100167515 *A. pisum*	Bioinformatics	1.19	983	*Myzus*, *A. pisum*, *R. padi*, *A. glycines*
LOC100167306 *A. pisum*	Bioinformatics	1.17	5666	*Myzus*, *R. padi*, *A. glycines*
ACYPI007464 *A. pisum*	Bioinformatics	1.17	13983	*Myzus*, *R. padi*, *A. glycines*
LOC100168723 *A. pisum*	Bioinformatics	1.16	1851	*Myzus*, *A. pisum*
Serine proteinase	Bioinformatics	1.15	937	*M. persicae*, *R. padi*, *A. pisum*
LOC100159010 *A. pisum*	Bioinformatics	1.14	5327	*Myzus*, *A. pisum*

In total we identified 430 orthologous groups in our RBBH network that contained a candidate effector based on our own analyses and several published candidate effector sets [11, 12, 20] out of a total of 31,361 groups. Out of these 430 groups, 49 were identified as being under positive selection (DN/DS > 1) (Table 2). In parallel, we selected a set of 35 sequences corresponding to genes not expected to be under positive selection based on their predicted conserved function in aphids (CDC42, EF1a, NADH-dehydrogenase, succinate dehydrogenase, TATA-box binding protein) for similar analyses. This generated 7 groups and DN/DS ratios <0.3, showing that none of these conserved genes were under positive selection. A further 390 groups corresponding to predicted EOG genes (Eukaryotic Orthologous Group) [29], also thought to be single copy, were subjected to DN/DS analysis. Three groups had DN/DS > 1 (values of 1.1, 1.1 and 1.4, 2dp) which we consider false positives, and correspond to 0.8 % of the EOG set. In contrast, 11.4 % of the putative effector containing clusters were found to have DN/DS > 1.0. However, we cannot rule out that some of these clusters actually represent gene duplicates rather than orthologs of single copy genes.

Putative effector clusters were checked to see if they contained single copy genes within the pea aphid genome. Of the 49 putative effector clusters under positive selection, 24 included an *A. pisum* sequence. To examine if these 24 aphid genes are represented by a single copy in the published *A. pisum* genome, their protein sequences were subjected to BLASTP searches against the predicted *A. pisum* protein set, excluding the expected self-matches. Three out of the 24 sequences returned hits when using a 70 % identity cut off, reflecting perhaps recent gene duplication, whilst 10 returned hits when using a 30 % identity cut-off, suggesting that at least 14 sequences are likely single copy.

The candidate effector group with the highest DN/DS ratio (4.17) included a protein of unknown function identified in the saliva of *M. persicae* (Table 2). The amino acid sequences in this cluster are mainly conserved in the N-terminal region. The C-terminal 51 amino acid region, which is predicted to be under the greatest positive selection pressure shows 21 amino acid differences between the genotype F and O (Additional file 11).

**Fig. 3:**
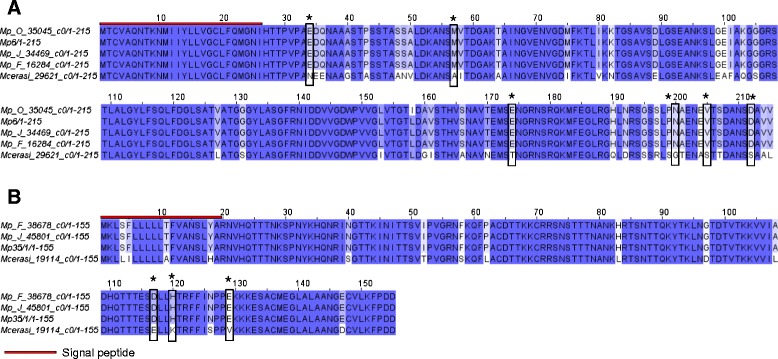
Orthologues sequences for previously identified effector were identified by reciprocal best blast hit analysis. DN/DS analysis identified clusters under positive selection pressure. **a** Sites which are most likely to be under positive selection (*P* > 0.95) are marked by *boxes* on the amino acid alignment for cluster containing Mp6 (DN/DN = 1.9). **b** Orthologues for Mp35 were identified as being under positive selection. Sites most likely to be under positive selection (*P* > 0.95) are marked with *boxes* (DN/DN = 3.0)

One group, containing *M. persicae* candidate effector Mp6 [11] was conserved within *M. persicae* genotypes, but divergent between *M. persicae* and *M. cerasi* (DN/DS ratio = 1.90) (Table 2; Fig. 3). We also identified a group, containing *M. persicae* candidate effector Mp35, with a DN/DS ratio of 3.0 that shows high conservation within *M. persicae*, but variation between the two different *Myzus* species (Table 2; Fig. 3). The sequences from these two species contain 32 nucleotide differences that correspond to 16 amino acid differences. This is also the case for Mp12 cluster, which is conserved in *M. persicae* but shows significant variation when compared to the pea aphid sequence (Table 2).

**Fig. 4:**
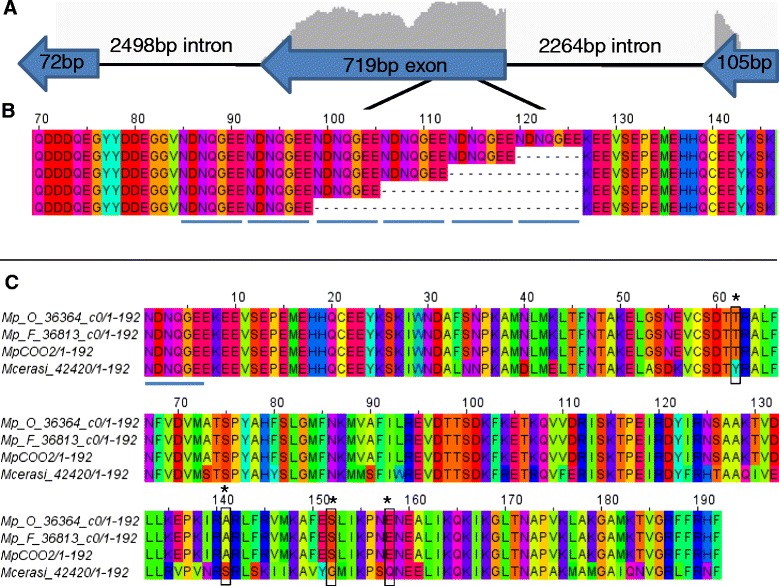
Analysis of C002 sequences identified. **a** MpC002 is a single copy gene encoded on the negative strand by three exons. The height of the *histogram-graph lines* represents the relative depth of RNA-seq coverage and therefore where the exon boundaries are. Within the 719 bp exon, variant transcripts, which contain 2–6 repeat motifs, are encoded. **b** The variant motif region for transcripts from *Myzus persicae* genotype O. **c** Reciprocal best blast hit analysis identified orthologues on a 1:1 ratio between the transcriptomes of C002. The C002 cluster was subjected to DN/DS analysis (with the multiple repeat region removed for analysis, this corresponded to the first 120 amino acids) was identified as being under selection pressure (DN/NS = 2.6). The sites most likely to be under selection pressure (*P* > 0.95) are marked on the alignment by *boxes*

For the RBBH group containing the aphid effector C002 [17] we removed the N-terminal repeat region, which is highly variable in length among different aphid species, prior to DN/DS analyses. The full-length *M. persicae* form of C002, here called MpC002, contains 5 repeat motifs (NDNQGEE, see Fig. 4(b)), which are important for virulence activity [13]. Our *M. persicae* transcriptome analyses identified variants with 2–6 repeat motifs. It is unclear whether this variation results from assembly artefacts or whether this is genuine variation. *MpC002* is a single copy gene encoded on the negative strand that contains a 105 bp exon, followed by a 2264 bp intron, followed by a 719 bp exon, followed by a 2498 bp intron, followed by a final 72 bp exon that is poorly supported by RNA-Seq data (Fig. 4). The repeat amino acid motifs are encoded within the 719 bp exon, with no evidence of any intron regions around this area for splicing to occur. *M. cerasi* C002 transcripts differ in this region, but again encode for a variable length repeat motif, composed of one NDDQGEV motif followed by four or five repeats of the NDNQGEV motif*.* No repeat motifs were found in *R. padi* C002, and this form was highly divergent from the *M. persicae* and *M. cerasi* forms and therefore did not fall within the C002 RBBH group. The DN/DS (2.6) value for the C002 cluster was based on variations seen between *M. persicae* and *M. cerasi*. The sequences corresponding to C002 within these species contained 81 nucleotide differences corresponding to 47 amino acid changes within the 159 amino acid region following the repeat motifs (Fig. 4(c)).

As mentioned above, sequences similar to *M. euphorbiae* effector Me10 [10] were among those most highly expressed in all species included within this study. Our RBBH and DN/DS analyses revealed the Me10-like sequences are under positive selection (Table 2).

**Fig. 5:**
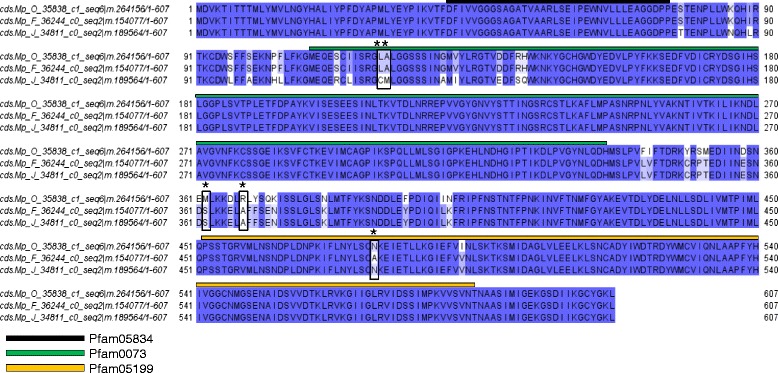
Sequences identified from the saliva of aphid species *Myzus persicae* via mass spectrometry analysis were identified as being under positive selection (DN/DS = 1.3). These were annotated as glucose dehydrogenase acceptor-like (similar to *Acyrthosiphon pisum*, gi|328715546). We found sites most likely to be under positive selection fall within GMC oxireductase PFAM domains (*P* > 0.95). These sites are marked on the alignment by boxes. Mutations in these domains may be important for co-evolution with host plants in a changing environment

We also identified several RBBH groups that contained sequences under positive selection among the different *M. persicae* genotypes (Table 2; Additional file 12). For example, Group_12749, with similarity to *A. pisum* XM_003246613.2, showed high levels of variation between *M. persicae* genotype F and the other two genotypes. Also, in Group_12369, which is annotated as a glucose dehydrogenase, we found variation between *M. persicae* genotypes. Interestingly we found sites under positive selection that fall within GMC oxireductase domains (Fig. 5). Mutations in these domains may be important for co-evolution with host plants in a changing environment. We also identified several pioneer effectors as being under positive selection. These were included in Group_12331, which contained *M. persicae* specific sequences and Group_6006, which only contains sequences from *M. persicae* and *M. cerasi* (Table 2). This highlights these pioneers are interesting sequences for further investigation.

Among the 49 RBBH groups we identified as potentially being under positive selection, 12 contained just the three *M. persicae* genotypes (Table 2; Additional file 12). In particular, we identified predicted effector variants within the transcriptome of genotype F that were different to those from the other genotypes. Compared to genotypes J and O, this F genotype is slow with regards to reproduction rates in several host plant species tested [25]. Whether this slow reproduction can be due to differences in aphid predicted effector repertoires is speculative, and remains to be further investigated.

## Conclusion

In conclusion we employed a combination of transcriptomics and saliva proteomics in order to identify and compare the putative effector repertoires from three aphid species. We have identified putative conserved effector sets, which are predicted to exhibit similar functions in different plant-aphid interactions. Such conserved effectors could be useful targets for the development of alternative control methods to provide broad range aphid control. Furthermore, we identified more diverse putative effector sets, which may be important for specific plant-aphid interactions and therefore in determining aphid host range.

## Methods

### Aphids stocks and material

Aphids were maintained in growth rooms at 18 °C with a 16 h light and 8 h dark period. *M. persicae* (genotype O, F and J) was maintained on potato (*Solanum tuberosum* cv. *Desiree*), *M. cerasi* was maintained on American Land Cress (*Barbarea verna*) and *R. padi* was maintained on barley (*Hordeum vulgare* cv. *Optic*). *M. persicae* (genotype O, F and J) were genotyped prior, during and after all samples were collected to ensure the integrity of the colony (Aphid lineages supplied and genotyped by Gaynor Malloch and Brian Fenton, The James Hutton Institute).

### RNA sample preparation and sequencing

Raw data is available at PRJEB9912 http://www.ebi.ac.uk/ena/data/view/PRJEB9912. Assemblies are also available through Aphidbase http://www.aphidbase.com/

Aphid heads, 100–200 per biological replicate, were dissected under a microscope in 1 % PBS. Aphid bodies were processed separately, 50–100 per biological replicate, by removing the head and removing any nymphs inside the aphid bodies. Dissected aphid samples were preserved in RNAlater (Sigma-Aldrich) until flash freezing in liquid nitrogen. Total RNA was extracted using a plant RNA extraction kit (Sigma-Aldrich), following the manufacturer’s instructions. We prepared three biological replicates for heads and bodies for each species and genotype. RNA quality was assessed using a Bioanalyzer (Agilent Technologies) and a Nanodrop (Thermo Scientific). RNA sequencing libraries were constructed with an insert size of 250 bp according to the TruSeq RNA protocol (Illumina), and sequenced at The Genome Analysis Centre, Norwich (http://www.tgac.ac.uk/) using Illumina-HiSeq 100 bp paired end sequencing.

### Filtering, quality control and assembly of RNA-seq data

An overview of our data analyses pipeline is shown in Fig. 1. The raw reads were assessed for quality before and after trimming using FastQC [48]. Raw reads were quality trimmed using Trimmomatic-0.32 [49], then assembled using Trinity with its *k*-mer coverage normalisation (version r20131110) [50]. CD-HIT (4.5.4) [51], with a threshold of 99 %, was used to reduce redundancy in the final assembly for *M. persicae* and *M. cerasi* datasets. *R. padi* was represented by 113,734,137 or 109,521,265 reads before and after trimming. In comparison *M. persicae* genotype O was represented by 137,205,488 or 124,984,399 reads before and after trimming, these number are representative of the other datasets. *R. padi* had the lowest number of reads generated. The read quantity could account for the smaller assembly for *R. padi.* The individual libraries of quality controlled reads where then mapped back to the post-CD HIT *de novo* assembly using Bowtie 1.0 [52] to assess digital expression. Normalised TMM-FPKM digital expression values and differential expression analysis was conducted using EdgeR [53], using *p* < 0.001 as a statistical threshold. Three biological replicate head and body libraries for each aphid species and genotype were prepared and sequenced. Following differential expression analysis and clustering of expression profiles, using EdgeR [53], an outlier head sample from *M. persicae* genotype F analysis (809_LIB4703_LDI4448_GATCAG_L008_R1 and R2) was removed from downstream analyses as it did not cluster with the other head samples.

### Prediction of coding sequences and annotation

For each component, loosely described by Trinity as a “gene”, the lowest expressing isoforms were removed as previously described [54]. This yielded the expressing transcripts per component. TransDecoder (version r20131117) [55] was used to predict the coding sequencing within transcripts using PfamA and PfamB definitions as a guide (release 27), transcripts that did not contain Pfam domains where also predicted by TransDecoder. The resulting coding sequences were annotated using Trinotate (version r_20131110) [50], HMMER (version 3.1b1) [56], Pfam (release 28) [54], SignalP (version 3.0) [57], TMHMM (version 2.0) [58], BLAST+ (version 2.2.30) [59], gene ontology [60], eggNOG (version 3.0) [61] and RNAmmer (version 1.2) [62]. A Galaxy pipeline [63] was used to identify putative secreted proteins by the presence of a signal peptide and the absence of a transmembrane domain [23]. Nuclear localisation was predicted using NoD (version 1.3b) [64] and cellular localisation was predicted using WoLF PSORT (last modified date 2006 Aug 31) [65], again within Galaxy [23]. Transcripts that were upregulated in the head tissue and were predicted to be secreted were classified as encoding putative effectors. Whereas, transcripts that were upregulated in body tissue were classified as encoding other secreted proteins. BLAST2GO (version 2.8, database September 2013) analysis was conducted using the online service (https://www.BLAST2go.com/b2ghome) [66]. Read mapping visualisation was performed using a combination of Tablet [67] and IGV [68].

The predicted unigenes were BLASTP searched against NCBI NR database (e-value 1e-5), BLAST+ version 2.2.30). The best hit was recorded for each sequence with the NCBI taxonomy ID, kingdom and genus.

To identify C002 transcripts which would previously have been collapsed (redundancy) in the assembly due to the use of CD-HIT, MIRAbait (version 4.0) [69] was used to identify reads that map onto the published C002 sequence (*k* = 25). The corresponding scaffold for C002 was identified using http://www.aphidbase.com/node_94263/Myzus-DB (Scaffold_246: August 2014). The identified reads were mapped using the splice-aware aligner TopHat (version 2.0.11) [70].

### Comparative transcriptomics

An overview of our data analyses pipeline is shown in Fig. 1. Predicted protein sets derived from the transcriptomes generated in this project as well as the transcriptomes of *A. glycines* (soybean aphid) [28] and *M. euphorbiae* (re-assembled for this project, as described for *R. padi*) [10], and the predicted proteins from the *A. pisum* (pea aphid) genome assembly V2.1 [26] and the *D. melanogaster* (fruit fly) genome release 5.55 [46] were clustered based on sequence similarity. Nucleotide coding sequences can be found in the following additional files: Additional files 13-17. All amino acid sequences were BLASTP searched against each other. The resulting BLAST network was then subjected to cluster analysis using MCL (version 12–135) [71]. Clustering analysis was conducted using BLAST threshold of 1e-35 with an MCL inflation value of I = 6. These values were chosen as they produced the greatest number of clusters representing the greatest number of sequences; increasingly strict BLAST e-value thresholds (e.g. 1e-36 to 1e-50) resulted in greater singleton clusters and sequences not represented in the cluster network.

In addition, we performed clustering of putative orthologous sequences as described in [72]. Briefly, putative 1:1 orthologues were identified using RBBH (Reciprocal Best BLAST Hit Analysis) between the amino acid sequences predicted from the 5 aphid transcript datasets generated in this project, the gene models for the pea aphid, and the soybean aphid (reassembled for this project), as described above. The thresholds for identification for putative 1:1 orthologues included using a minimum threshold of 70 % identity and 50 % query coverage. Tied top scoring BLAST hits (as might be expected with recent gene duplications) were rejected by the RBBH script, which identified pairs only https://github.com/peterjc/galaxy_blast/tree/master/tools/blast_rbh [73]. This analysis is limited by the availability of transcriptome data only, and did not take into consideration possible gene duplication, which is common in aphid genomes. Once we identified RBBH-partner sequences, a network was generated using MCL from the RBBH data, resulting in RBBH-clusters. The RBBH data was passed into MCL as an abc file: query, hit, e-value. We calculated the DN/DS values for each cluster that contained a predicted effector protein based on our work as well as several published studies [11, 12, 20]. To calculate the DN/DS ratio for a cluster of orthologues, the sequences were aligned using MUSCLE (version 3.8.31) [74], then the nucleotide sequences were back-translated onto the alignments (https://github.com/peterjc/pico_galaxy/tree/master/tools/align_back_trans) [75]. These were then manually altered using Jalview [76] by removing non-consensus, possibly miss-predicted 5′ and 3′ regions based on either pea aphid genome annotations and/or consensus sequences, where appropriate. Indel regions were also removed, as was the repeat motif region in putative effector C002. Modified alignments were subjected to DN/NS analysis using CodonPhyml (version 1.0) [77]. The trees generated by CodonPhyml were then used by PAML (version 4.8) (Codeml) [78] to identify the site most likely to be under selection pressure.

### Phylogenetic analysis

Single copy orthologous genes identified and analysed by Misof et al. [29] were used as a basis for phylogenetic analysis. RBBH analysis (as described above) between the transcriptomes and the pea aphid protein set identified orthologous sequences. This was used to identify orthologues from the transcriptomes to those genes used for the pea aphid sequences in Misof et al. [29]. Only 712 out of 1478 orthologues from the genes used by Misof et al. [29] were identified. We only included genes represented by 4 or more of the aphid species used for analyses, which amounted to 71 genes. The amino acid sequences corresponding to these genes were aligned using MUSCLE (with refine option) and the nucleotide sequences were back-translated to the alignment. The 71 aligned orthologous genes from Misof et al. [29] were concatenated with the back-translated alignments (as described above) and subjected to further alignment. MEGA6 [79] was used for phylogenetic analysis (Maximum likelihood 150 boot straps).

### Proteomic analysis

Aphids were transferred to a feeding chamber as described in Harmel et al. [22]. We used a diet similar to phloem sap (15 % sucrose, 100 mM L-serine, 100 mM L-methionine and 100 mM L-aspartic acid with a pH of 7.2 (KOH)) [18]. Approximately 60,000 aphids per species per genotype were fed on this artificial diet system. Diet/saliva mixes were collected 24 h after exposing the aphids to the diet. Samples were then concentrated using protein concentration columns with a 9KDa molecular weight cut off (Thermo Scientific). Concentrated samples were run on SDS-PAGE gels and lanes were divided in three parts of equal size. Gel slices were processed and subjected to LC-MS/MS analyses using a RSLCnano UHPLC system coupled to a LTQ Orbitrap Velos Pro MS system (Thermo Scientific) at the University of Dundee Fingerprints Facility. We also analyzed the samples containing peptide and proteins <9 kDa, the flow through from the concentration columns, by LC-MS/MS. MASCOT software (version 2.4.1) searches against our transcriptome datasets as well as NCBI NR were used for peptide identification.

## Additional files

Additional file 1:
**Nucleotide alignment of aphid EOG sequences for phylogenetic analysis.** (FASTA 1597 kb) Additional file 2:
**Annotation of the 100 most abundant transcript’s coding sequence for each of the aphids investigated.** Top BLAST hit annotation for each of the identified coding sequences. (XLSX 27 kb) Additional file 3:
**Annotation and amino acid sequences for predicted effectors based on RNA-seq and bioinformatics analyses.** Also includes a list of previously identified putative effectors and corresponding sequences identified in this study (sheet 2). (XLSX 351 kb) Additional file 4:
**Expression profiles of constitutively expressed genes.** TMM-FPKM expression profile for transcripts constitutively expressed. (XLSX 15 kb) Additional file 5:
**GO enrichment analysis for transcripts upregulated in heads, bodies predicted to encode secreted proteins for each species.** (PPTX 3205 kb) Additional file 6:
**GO enrichment analysis data for all species.** (TXT 9449 kb) Additional file 7:
**Proteins identified in aphid saliva using LC-MS/MS.** Proteins identified in aphid saliva using LC-MS/MS when searching against the *de novo transcriptomes* and against NCBI NR database. The corresponding sequences of the proteins identified, and the raw LC-MS/MS coverage data for each dataset investigated. (XLSX 99 kb) Additional file 8:
**Graphical representation of the expression profiles of transcripts corresponding to candidate effectors identified by proteomics based on their digital RNA-seq expression profiles.** The blue line represents the mean for all expression profiles and the green line represents +/− two standard deviations. (PDF 4 kb) Additional file 9:
**Putative conserved effectors.** List of conserved sequence clusters that contain candidate effectors identified in this study by transcriptomics or proteomics, or previously identified [22, 23, 50]. (XLS 51 kb) Additional file 10:
**Clusters.** Resulting clusters (one line per cluster) from aphid sequences subjected to MCL clustering analysis (BLAST-MCL-clustering). Another clustering method was used to identify 1:1 orthologues by using reciprocal best BLAST hit analysis of all the datasets, resulting in an RBBH-orthologues network (one line per cluster). Putative pioneer effectors were identified and their sequences are reported. (XLSX 4051 kb) Additional file 11:
**Amino acid alignment for cluster_1269 which contained a proteomic identified sequence.** This putative effector containing cluster had the greatest DN/DS ratio of all the effector containing clusters (DN/DS = 4.17). The sites most likely to be under selection pressure (*P* > 0.95) are marked on the alignment by boxes asterisks. (PPTX 142 kb) Additional file 12:
**Nucleotide alignments.** Clusters of interest identified from the 1:1 orthologues clustering method predicted to be under selection pressure based on DN/DS analysis. (ZIP 50 kb) Additional file 13:
**Predicted nucleotide coding sequences for** ***Myzus persicae*** **genotype O.** (GZ 12701 kb) Additional file 14:
**Predicted nucleotide coding sequences for** ***Myzus persicae*** **genotype F.** (GZ 12050 kb) Additional file 15:
**Predicted nucleotide coding sequences for** ***Myzus persicae*** **genotype J.** (GZ 11406 kb) Additional file 16:
**Predicted nucleotide coding sequences for** ***Myzus cerasi.*** (GZ 13367 kb) Additional file 17:
**Predicted nucleotide coding sequences for** ***Rhopalosiphum padi*** **and** ***Aphis glycines***. (GZ 8032 kb)
