# Supplementary material for: Comparative transcriptomics and proteomics of three different aphid species identifies core and diverse effector sets
Source: BMC Genomics. 2016 Mar 2;17:172. doi: 10.1186/s12864-016-2496-6 (PMC4776380; doi:10.1186/s12864-016-2496-6)
Supplement: Additional file 5: — GO enrichment analysis for transcripts upregulated in heads, bodies predicted to encode secreted proteins for each species. (PPTX 3205 kb) [file 12864_2016_2496_MOESM5_ESM.pptx]

## Slide 1
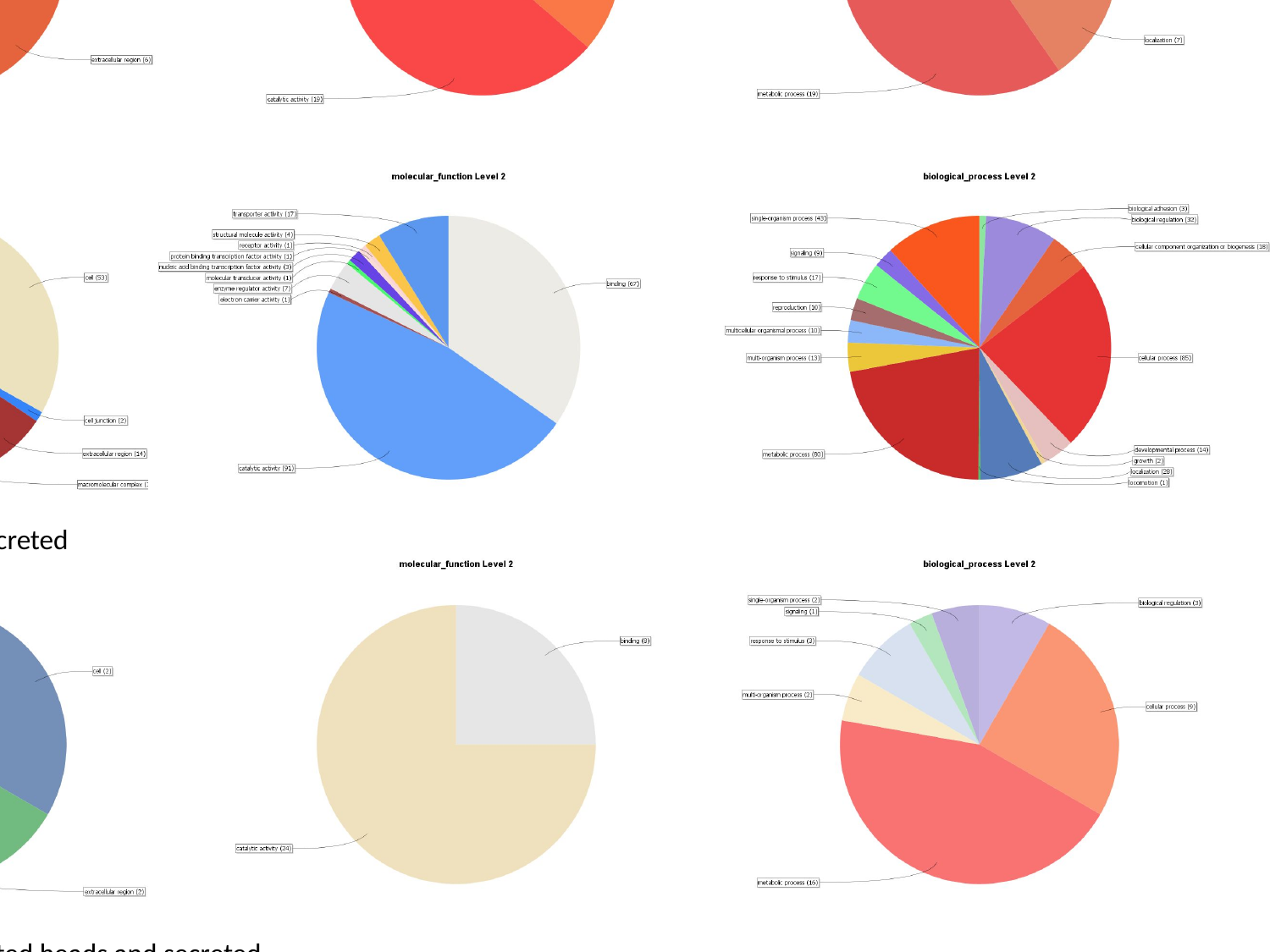

M. cerasi
Up regulated heads
Up regulated heads and secreted
Upregulated bodies
Upregulated bodies and secreted
GO enrichment: Up regulated heads and secreted

## Slide 2
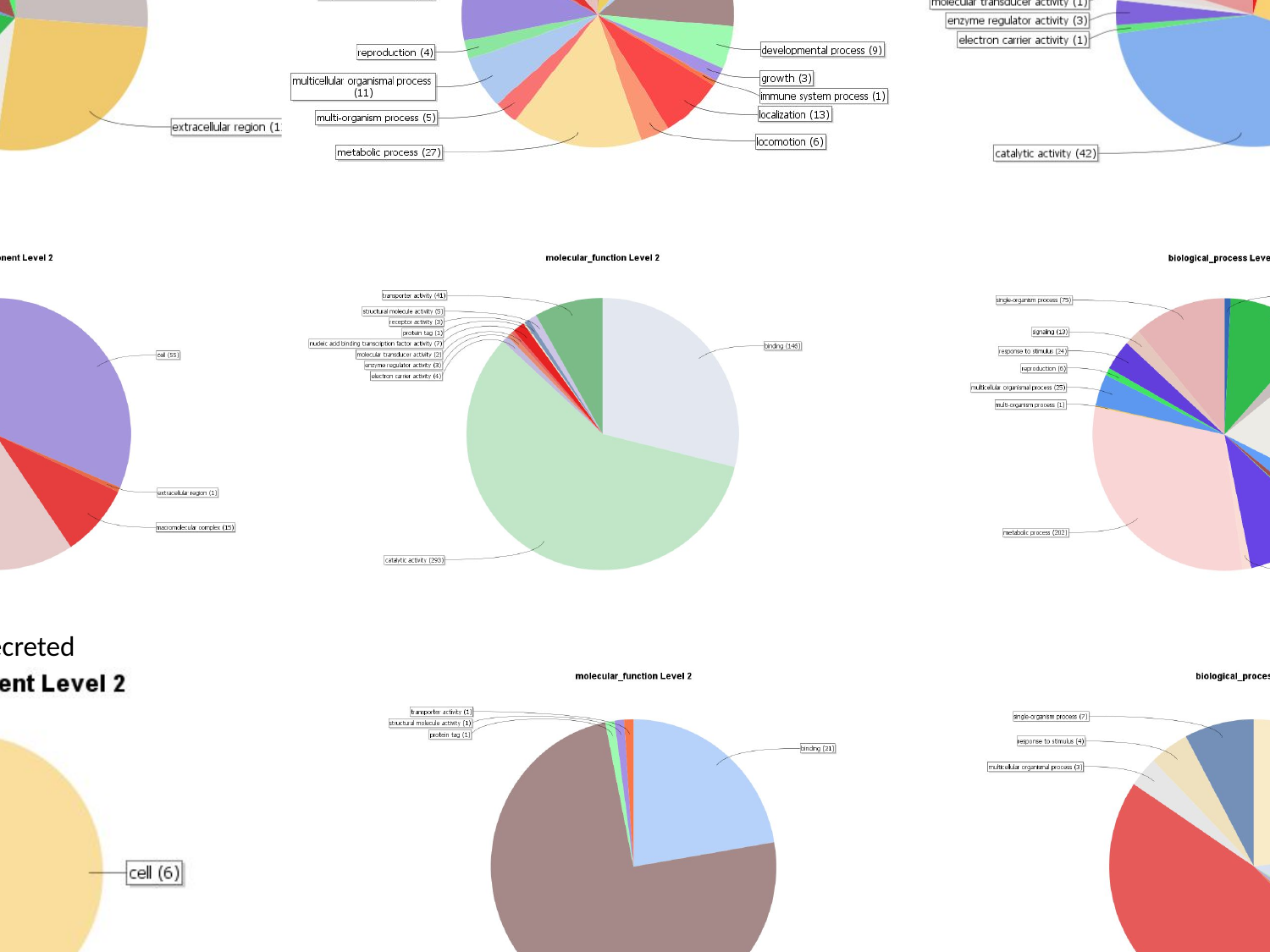

M. persicae genotype O
Up regulated heads
Up regulated heads and secreted
Upregulated bodies
Upregulated bodies and secreted
GO enrichment: Up regulated heads and secreted

## Slide 3
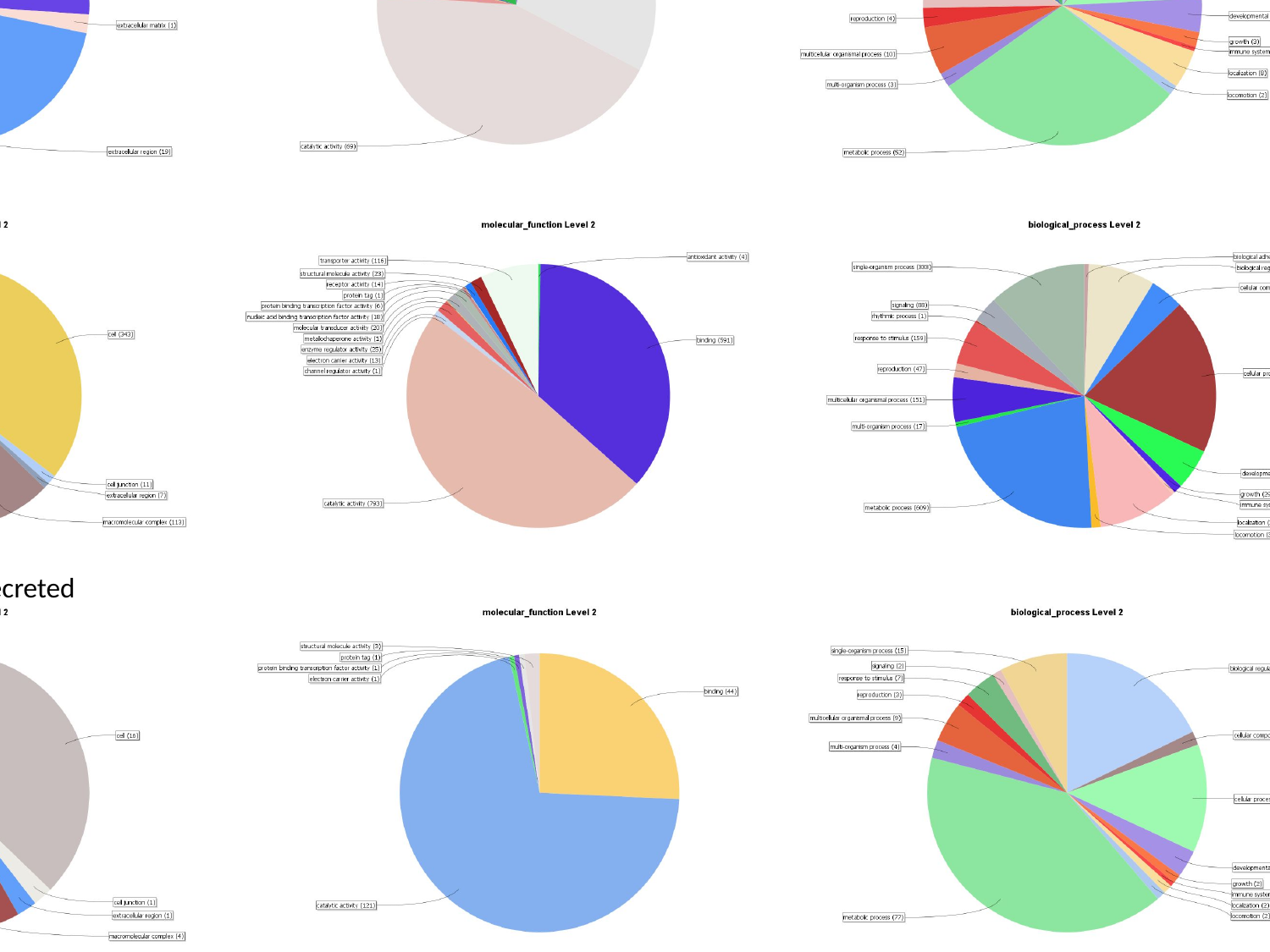

M. persicae genotype J
Up regulated heads
Up regulated heads and secreted
Upregulated bodies
Upregulated bodies and secreted
GO enrichment: Up regulated heads and secreted

## Slide 4
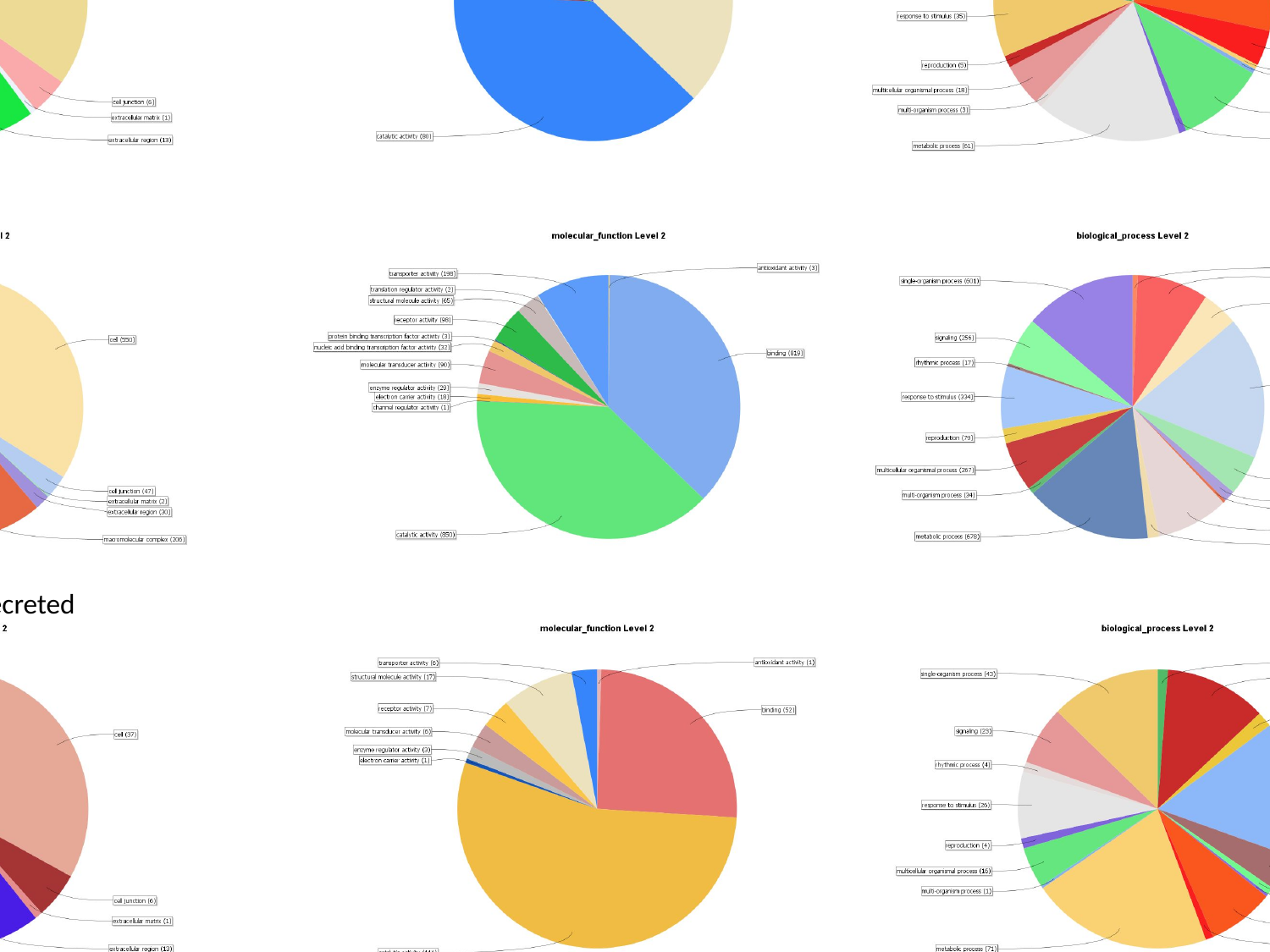

M. persicae genotype F
Up regulated heads
Up regulated heads and secreted
Upregulated bodies
Upregulated bodies and secreted
GO enrichment: Up regulated heads and secreted

## Slide 5
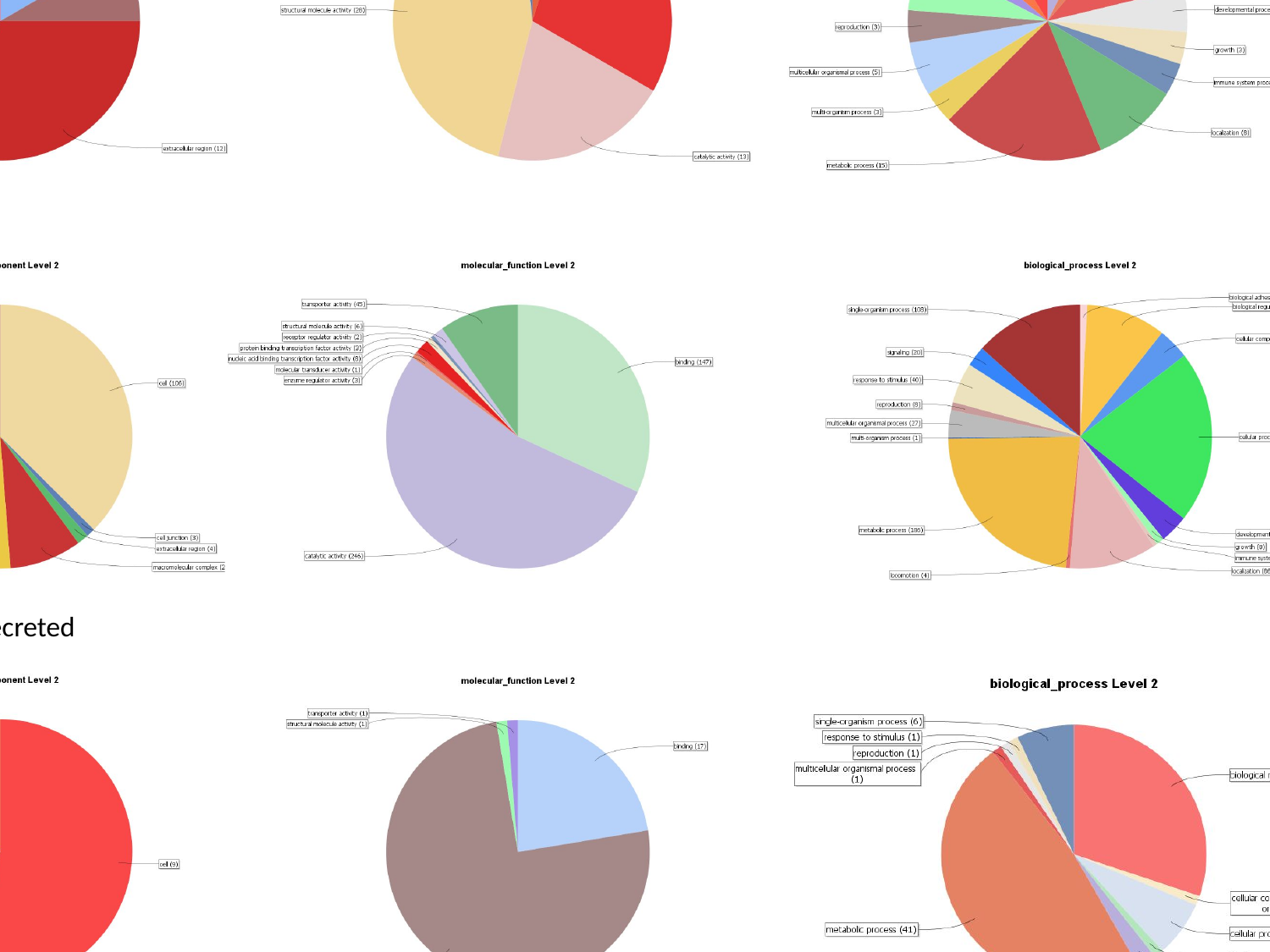

R.padi
Up regulated heads
Up regulated heads and secreted
Upregulated bodies
Upregulated bodies and secreted
GO enrichment: Up regulated heads and secreted
