# Supplementary figures and images for: Comparative transcriptomics and proteomics of three different aphid species identifies core and diverse effector sets
Source: BMC Genomics. 2016 Mar 2;17:172. doi: 10.1186/s12864-016-2496-6 (PMC4776380; doi:10.1186/s12864-016-2496-6)

# Proteomic Identified proteins

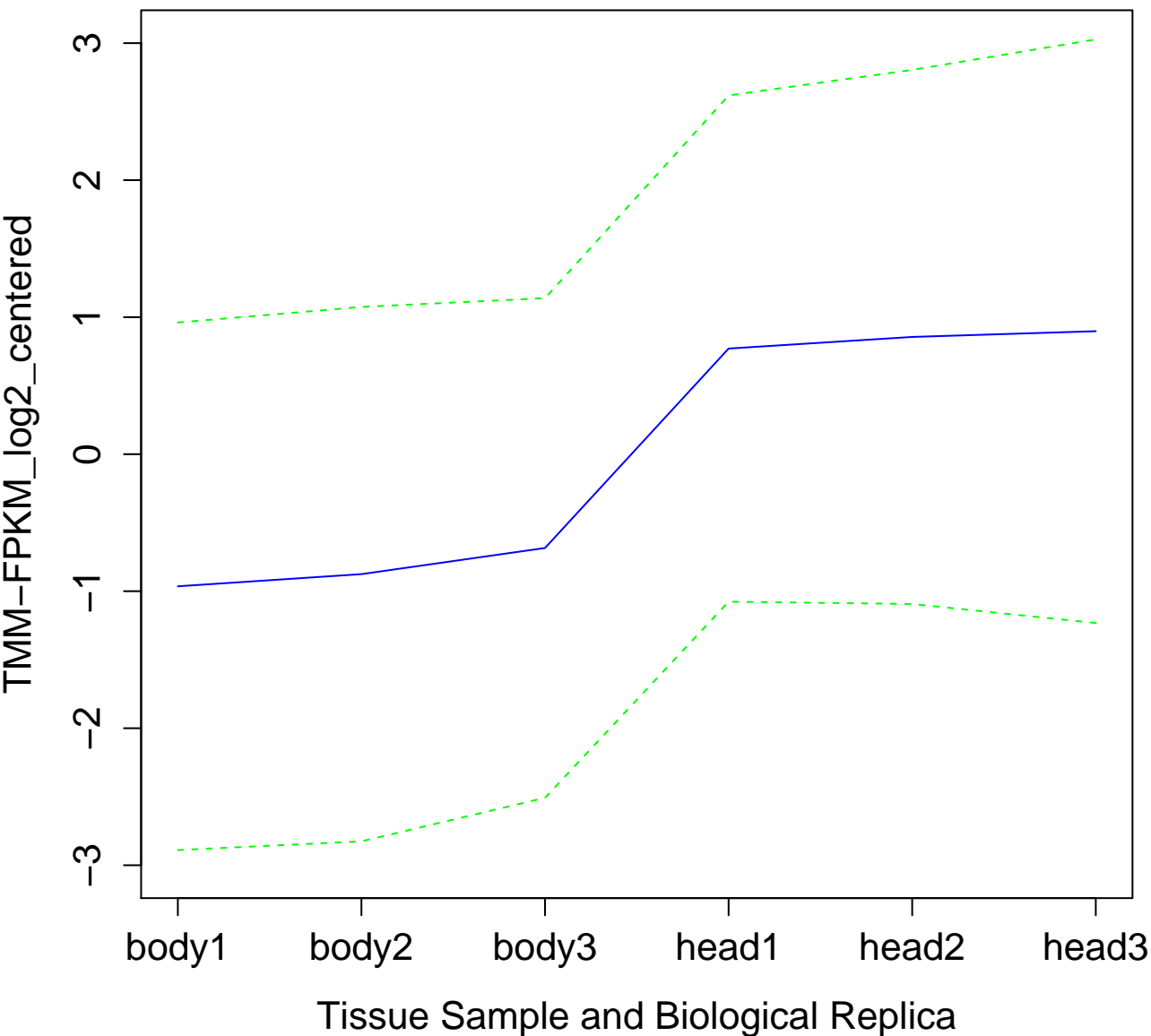

Supplement: Additional file 8: — Graphical representation of the expression profiles of transcripts corresponding to candidate effectors identified by proteomics based on their digital RNA-seq expression profiles. The blue line represents the mean for all expression profiles and the green line represents +/− two standard deviations. (PDF 4 kb) [file 12864_2016_2496_MOESM8_ESM.pdf]

## Slide 1
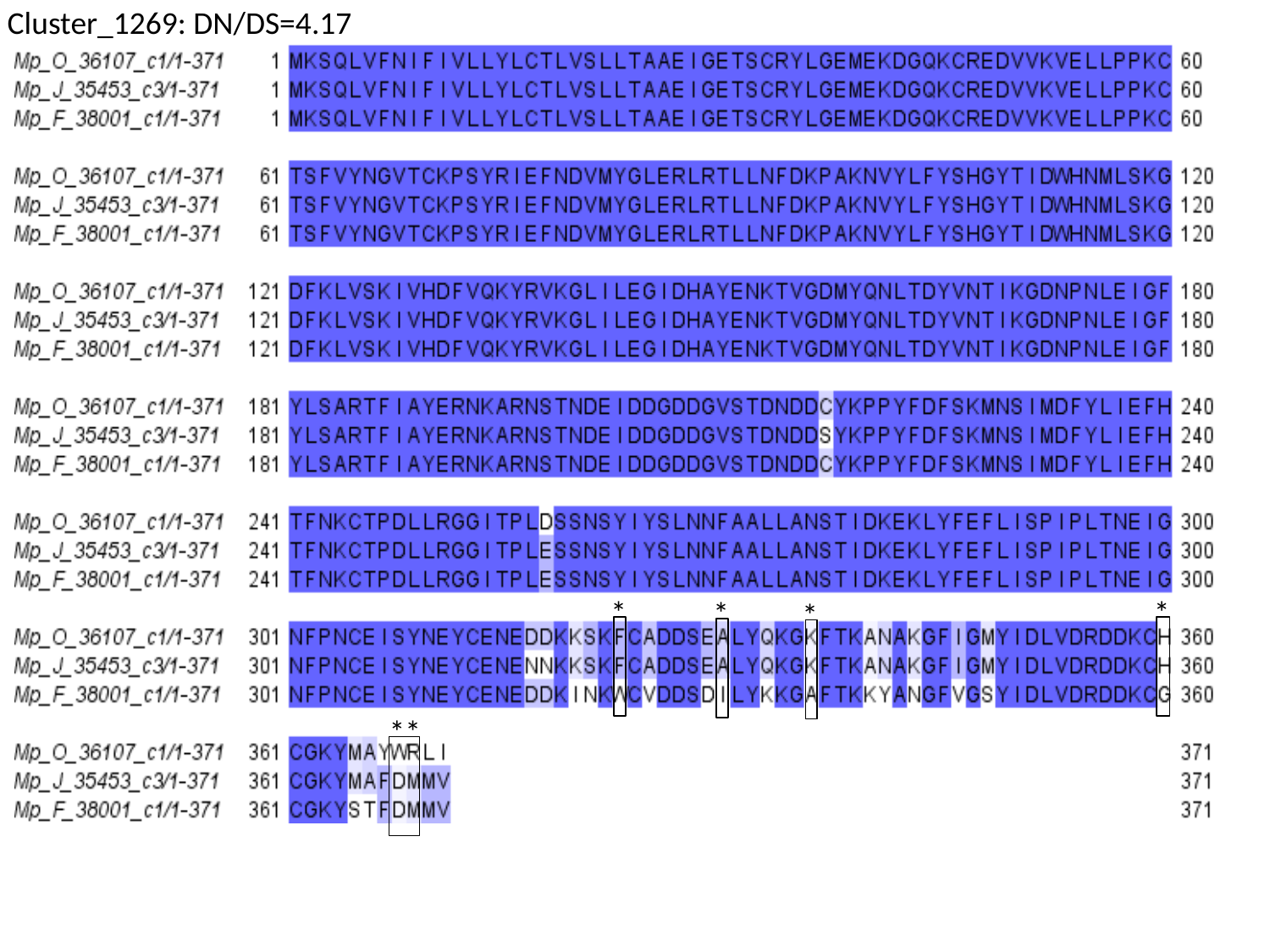

Cluster_1269: DN/DS=4.17
Cluster_1269: DN/DS=4.17
*
*
*
*
**

Supplement: Additional file 11: — Amino acid alignment for cluster_1269 which contained a proteomic identified sequence. This putative effector containing cluster had the greatest DN/DS ratio of all the effector containing clusters (DN/DS = 4.17). The sites most likely to be under selection pressure (P > 0.95) are marked on the alignment by boxes asterisks. (PPTX 142 kb) [file 12864_2016_2496_MOESM11_ESM.pptx]
